# Spinal muscular atrophy with respiratory distress type 1: Clinical phenotypes, molecular pathogenesis and therapeutic insights

**DOI:** 10.1111/jcmm.14874

**Published:** 2019-12-04

**Authors:** Matteo Saladini, Monica Nizzardo, Alessandra Govoni, Michela Taiana, Nereo Bresolin, Giacomo P. Comi, Stefania Corti

**Affiliations:** ^1^ Dino Ferrari Centre Neuroscience Section Department of Pathophysiology and Transplantation (DEPT) University of Milan Milan Italy; ^2^ Foundation IRCCS Ca' Granda Ospedale Maggiore Policlinico Neurology Unit Milan Italy; ^3^ Foundation IRCCS Ca' Granda Ospedale Maggiore Policlinico, Neuromuscular and rare diseases unit Milan Italy

**Keywords:** gene therapy, IGHMBP2, motor neuron disease, Spinal muscular atrophy with respiratory distress type 1

## Abstract

Spinal muscular atrophy with respiratory distress type 1 (SMARD1) is a rare autosomal recessive neuromuscular disorder caused by mutations in the *IGHMBP2* gene, which encodes immunoglobulin μ‐binding protein 2, leading to progressive spinal motor neuron degeneration. We review the data available in the literature about SMARD1. The vast majority of patients show an onset of typical symptoms in the first year of life. The main clinical features are distal muscular atrophy and diaphragmatic palsy, for which permanent supportive ventilation is required. No effective treatment is available yet, but novel therapeutic approaches, such as gene therapy, have shown encouraging results in preclinical settings and thus represent possible methods for treating SMARD1. Significant advancements in the understanding of both the SMARD1 clinical spectrum and its molecular mechanisms have allowed the rapid translation of preclinical therapeutic strategies to human patients to improve the poor prognosis of this devastating disease.

## 1 INTRODUCTION

Autosomal recessive spinal muscular atrophy with respiratory distress type 1 (SMARD1) is a form of spinal muscular atrophy with severe diaphragmatic involvement that causes respiratory distress. This condition is due to autosomal recessive mutations in the *IGHMBP2* gene, which is located on chromosome 11q13.2‐q13.4.^1^, ^2^ Mellins, considering this mutation a variant of spinal muscular atrophy (SMA) 5q with respiratory onset, provided the first description of this condition in 1974, and it was not recognized as a separate clinical entity until 1996.^3^, ^4^ The actual prevalence of SMARD1 is unknown, but diaphragmatic paralysis is observed in approximately 1% of patients with an early onset of the clinical features of spinal muscle atrophy and an estimated incidence of 1/100 000.^5^ The main clinical feature is the onset of respiratory distress requiring mechanical ventilation between the ages of 6 weeks and 6 months. The clinical symptoms rapidly progress in the first years of life, with distal limb muscular atrophy extending to proximal regions. The overall prognosis is poor, and progressive autonomic nervous system dysfunction also develops in association with the progressive worsening of motor functions in affected children. In fact, there are no approved treatments for SMARD1.^6^

## 2 CLINICAL FEATURES

### 2.1 Neonatal features

There is no specific neonatal clinical marker of this disease, although intrauterine growth retardation and premature birth are very common.^6^ The majority of affected children present with nonspecific symptoms, such as weak cry, hypotonia, feeding problems, weak suckling and recurrent respiratory infections, in the first weeks of life.^5^, ^7^ Congenital foot malformations caused by distal muscle development defects and by the deposit of fatty pads in the proximal phalanges are also frequently found.^8^

### 2.2 Respiratory distress

Respiratory distress is usually the presenting clinical symptom and occurs between 6 weeks and 6 months of age as a consequence of the development of neurogenic diaphragmatic palsy. The presentation of respiratory distress is characterized by inspiratory stridor, weak cry, recurrent bronchopneumonia and trouble eating. This condition is almost always life‐threatening in the absence of medical intervention; thus, a “pro‐life decision” is often required before the diagnosis is genetically confirmed.^6^, ^8^, ^9^, ^10^ Unlike SMA patients, who exhibit a bell‐shaped chest and paradoxical respiration as a consequence of intercostal muscle palsy, SMARD1 patients have a normal‐shaped thorax because the defect mainly involves the diaphragm.^5^, ^6^, ^7^, ^8^ Chest X‐ray, which can show the characteristic eventration (the abnormal elevation) of the right or, less frequently, both hemidiaphragms, which is considered a highly suggestive sign of SMARD1, plays a core role in the diagnostic pathway. The confirmation of paralysis can be achieved by performing a chest ultrasound, diaphragmatic electromyography or fluoroscopy.^5^, ^6^, ^7^, ^8^

### 2.3 Neuromuscular features

The degeneration of the phrenic nerve is accompanied by the progressive wasting of the distal muscles of the limbs; the lower limbs are affected earlier than the upper limbs, and the proximal muscles become affected along with the progression of the disease.^6^, ^10^ The natural history of SMARD1 leads to complete paralysis of the four limbs, with an absence of deep tendon reflexes usually after the first year of life and the development of rachis malformations, such as kyphoscoliosis. The clinical features seem to progress most rapidly in the first two years of life, followed by a stabilization of the pattern and sometimes a mild improvement of some functions, such as respiratory activity and muscle strength, most likely due to the regeneration of some muscle fibres.^6^, ^8^, ^10^

Regarding neurological assessment, motor development milestones and communication skills, in particular those specified in the semiquantitative scoring system by Eckart et al,^10^ have been proposed as outcome measures Table 1. The authors proposed applying this score monthly in the first year of life and then yearly during follow‐up.

**Table 1:** Semiquantitative original scoring system, from Eckart and colleagues

Eckart's semiquantitative scoring system for SMARD1		Y/N
1	Onset of mechanical ventilation	
2	Onset of muscle weakness	
3	Remaining antigravity movements in arms	
4	Remaining antigravity movements in legs	
5	Sitting without support	
6	Holding the head upright while in sitting position	
7	Reduction in facial expression	
8	Ability to speak	
9	Freedom from cerebral seizures	
10	Dysfunction of the autonomic nervous system	

### 2.4 Central and autonomic nervous system abnormalities

Cranial nerves are frequently involved in the natural course of SMARD1, although not as a presenting feature; mimicking pathologies, such as muscle weakness and tongue fasciculations, have been reported as signs of hypoglossal nerve paralysis, while the oculomotor nerves are usually spared. Epileptic seizures have also been reported, but these disorders do not seem to be aetiologically related to the disease.^8^ Autonomic nervous system involvement is common in SMARD1 patients and manifests mainly as bladder incontinence, urinary retention with the need for catheterization, excessive sweating, constipation and cardiac arrhythmia.^6^, ^8^ In some cases, autonomic dysfunction can be the prominent feature of the clinical course, such as in the case of a Japanese girl affected with genetically confirmed SMARD1 who presented with catastrophic autonomic crisis with cardiac collapse, as reported by Nomura and colleagues.^11^

## 3 DIAGNOSIS

SMARD1 patients typically present with phrenic nerve palsy between the ages of six weeks and six months, and motor neuron degeneration primarily affects the distal muscles, particularly those of the lower extremities.^6^ According to a cohort study enrolling 141 patients by Guenther and colleagues, the combination of these features is present in 86% of patients with *IGHMBP2* mutations. Moreover, the onset of respiratory distress between six weeks to six months of age combined with preterm birth or right diaphragm eventration seems to predict the disease with a sensitivity of 98% and a specificity of 92%.^12^ Conversely, the congenital onset of respiratory distress is not typical in SMARD1 and is consistent with other more common diagnoses, the most common of which are SMA1, congenital myotonic dystrophy type 1 (cDM1), early‐onset myopathy, areflexia, respiratory distress and dysphagia (EMARDD), and congenital myasthenia gravis.^8^, ^10^, ^13^, ^14^ The current practice is to follow the diagnostic criteria for SMARD1 provided by Pitt and colleagues in 2003, which are reported in Table 2.

**Table 2:** The diagnostic criteria for SMARD1, which may permit its effective distinction from other similar conditions^15^

Clinical criteria	Histopathological criteria	EMG criteria
Low birthweight below the 3rd centile	Reduction of myelinated fibre size in sural nerve biopsies	Evidence of acute or chronic distal denervation
Onset of symptoms within the first 3 months	Minimal evidence of ongoing myelinated fibre degeneration in biopsies taken up to 3 ± 4 months	Evidence of severe slowing (<70% of lower limit of normality in one or more nerves (motor or sensory))
Diaphragmatic weakness either unilaterally or bilaterally	No evidence of regeneration or of demyelination that might account for the change in fibre size.	
Ventilator dependence within less than one month of onset with an inability to wean		
Absence of other dysmorphology or other conditions		

In a recent retrospective analysis of 22 cases, the authors confirmed the predictive effects of Pitt's criteria for the diagnosis of SMARD1, but at least 4 of the patients had normal chest X‐rays at the onset of respiratory distress. Based on this evidence, these authors hypothesized that genetic tests for *IGHMBP2* could be performed even before the onset of diaphragm eventration. This hypothesis is justified by the observation that 28.6% of the cases involved intrauterine growth restriction (IUGR) and distal deformities at birth and suggests that clinicians should be alerted in each case of a premature newborn with symmetrical IUGR under the 10th percentile and respiratory warning signs (weak cry, stridor and feeding difficulties), even without positive chest X‐ray findings.^16^

## 4 NATURAL COURSE AND PROGNOSIS

SMARD1 is a chronic, congenital and untreatable disease, and much progress is still needed to improve the outcomes and quality of the lives of affected patients. In 2012, Eckart and colleagues reported a broad analysis of the natural course of life in 11 SMARD1 cases, in which these authors observed that the loss of independent breathing and the onset of muscle weakness took place between 2 and 9 months of age, prompting an immediate pro‐life decision. By 9 months, all of the patients needed mechanical ventilation to survive, and only one of the patients, who was able to regain the ability to breathe independently through the use of accessory muscles for 12 hours per day, could be weaned from mechanical ventilation during the observation period, which was on average 7.8 years. None of the patients were able to reach and maintain a standing position or walk and thus needed wheelchair mobilization. Autonomic nervous system failure was present in all of the patients, the most recent of which developed this condition beyond the age of 9 years. Seven out of the 11 patients underwent echocardiographic screening, with no evidence of any structural cardiac abnormality being found.

At the end of the observational period, 7 out of the 11 children exhibited normal facial expressions, four patients could sit and hold their heads, six were able to speak through a speech cannula or a leak in their tracheal cannula, and five patients remained seizure‐free. All of these patients, with the exception of one, lived at home and could have normal social interactions with other children.

This study demonstrated marked differences in the severity and rate of progression of symptoms in patients with improved severity scores between 1 and 3 years of age. Moreover, the authors observed that an overall higher score at the age of 3 years was usually associated with a slower progression of the disease and milder symptoms.

The overall survival rate of SMARD1 without artificial ventilation, with a mean age of death of 9 months, is very poor.^17^ Evidence suggests that there are three different forms of SMARD1 that vary based on the age of onset: an early‐onset form, which usually appears before three months of age and is very severe, a classical onset form; and a very rare late‐onset form, which is characterized by milder symptoms and a slower progression compared with that of the classical onset form and affects patients usually after the age of 12 months.^17^ As of now, the oldest affected patient to be described was a 21‐year‐old girl. After having achieved the ability to crawl and stand with support, she was clinically diagnosed at the age of 16 months after presenting with mild right hemidiaphragm eventration upon chest X‐ray and a clinical picture of cough, wheeze and signs of respiratory distress. Successive analyses confirmed the diagnosis of a late‐onset form of SMARD1. This patient uses a ventilator only at night, and her cognitive skills are unaffected.^18^

The death of the great majority of patients is caused by the development of respiratory failure or by complications related to mechanical ventilation, such as sepsis or pneumonia. The average life expectancy is very difficult to estimate because it depends on the severity of respiratory involvement, provided treatments and susceptibility to ventilator‐associated complications.^10^

## 5 PATHOGENESIS

SMARD1 is caused by autosomal recessive mutations in *IGHMBP2* on chromosome 11q13.2‐q13.4.^1^, ^2^ Human *IGHMBP2* is composed of 15 exons and encodes immunoglobulin µ‐binding protein 2, a protein that comprises 993 amino acids (109 149 Da) and whose function is not still understood. The gene product is an ATPase/helicase of the SF1 superfamily that is ubiquitously expressed, although this protein is expressed mainly in the cytoplasm, where it associates with ribosomes.^19^ The *IGHMBP2* gene harbours four domains: an ATPase domain, a single‐stranded nucleic acid‐binding R3H domain, a DEXDc domain and an AN1‐type zinc finger motif. Biochemical characterization has determined that *IGHMBP2* is an ATP‐dependent 5’→3’ DNA/RNA helicase. Most SMARD1‐causing missense mutations have been found in the helicase domain, which contains two recA‐like subdomains and at least seven highly conserved motifs of amino acids, suggesting that this domain may play a central role in the pathogenesis of the disease.^9^, ^20^ Further investigations have revealed that the synergy of the helicase domain with the R3H domain leads to an enhanced RNA binding affinity, thus potentiating its ATPase activity.^21^

The IGHMBP2 protein comprises a DNA/RNA helicase domain, an R3H domain, a zinc finger domain, a DEXDc domain and an AN1‐type zinc finger motif. Based on sequence homology, this protein has been classified as part of the UPF1‐like group of helicase superfamily 1 (SF1). The full extent of the function of the IGHMBP2 protein is not yet known, although this protein may take part in several different cellular functions, such as pre‐mRNA processing, immunoglobulin class switching, and the regulation of DNA replication and the interaction with the TATA‐binding protein. Furthermore, IGHMBP2 acts as an ATP‐dependent helicase and may play a role in ribosomal translation and biogenesis.^2^, ^21^

The fact that the great majority of known *IGHMBP2* mutations are located in the helicase and R3H domains has led the authors to believe that these domains are essential to the function of the protein and has sparked interest in correlating the level of ATPase activity to the severity of the clinical phenotype. In Guenther et al^9^ was the first group to study the protein expression levels in EBV‐immortalized lymphoblastoid cell lines (LCLs) from SMARD1 patients and their parents. These authors found that patients with a milder juvenile form of SMARD1 had higher levels of protein expression than those of severe SMARD1‐affected patients and that their parents expressed proteins at a level that was 75% of that exhibited by healthy controls. Moreover, these authors observed that the mRNA levels were not correlated with the amount of functional protein and thus concluded that SMARD1 pathogenesis may be related to reduced translation or protein degradation rather than diminished mRNA levels (Guenther et al^9^). Further evidence is required to better confirm the existence of this correlation because it may be very important in the development of therapeutic strategies for SMARD1.

## 6 *IGHMBP2* MUTATION IN CHARCOT‐MARIE‐TOOTH DISEASE

Charcot‐Marie‐Tooth disease (CMT) is a spectrum of multiple hereditary syndromes causing the progressive length‐dependent degeneration of peripheral sensory and/or motor fibres. CMT has an estimated prevalence of 1:2500, and its aetiology is related to more than 80 different genes.^22^ The diverse phenotypic and electrophysiological symptoms lead to the classification of CMT by type (type 1 and type 2).^22^, ^23^ In 2012, the Cottenie group discovered an *IGHMBP2* mutation in two consanguineous English patients affected by CMT type 2S disease (CMT2S) through a combination of genome sequencing and linkage analysis. Pathological studies showed some differences between the features of these patients and the typical CMT2 pattern observed in cases involving mutations in *MFN2*. CMT2 cases involving mutations in *IGHMBP2* tend to have a milder involvement of large myelinated fibres, which is almost absent in CMT2 cases involving mutations in *MFN2*, which show no involvement of small fibres. Moreover, ultrastructural analysis showed few actively degenerating axons, which are very rare in patients with *MFN2* mutations.^24^ In recent years, many other similar cases have been described, and to date, 21 recessive *IGHMBP2* variants have been associated with a CMT2S phenotype.^25^, ^26^

The observation that CMT2S and SMARD1 are caused by different mutations in the same gene led the authors to investigate the cause of the differences in clinical phenotype. Mutations in SMARD1 are predominately missense and located in the helicase domain, while in CMT2, the mutations were mainly a combination of a nonsense mutation in the 5′ region of the gene with truncating frameshift, missense or homozygous frameshift mutations in the last exon. This different combination of mutations in the two diseases leads to a different amount of residual protein. In fact, the quantification of protein levels in fibroblasts and lymphoblastoid cell lines demonstrated that CMT2S patients have significantly higher IGHMBP2 protein levels than SMARD1 patients, although a defective truncated protein was found in many cases. Furthermore, the mRNA levels were normal or higher than normal in both cell lines, suggesting that posttranslational degradation mechanisms may be responsible for the absence of the IGHMBP2 protein and a correlation between the phenotype and the quantity of residual protein has therefore been hypothesized.^24^ Moreover, the function of IGHMBP2 is similar to that of GARS, AARS, HSPB1 and HSPB8, which have all been associated with both CMT and distal hereditary motor neuronopathy (dHMN) phenotypes. This evidence suggests that dysfunctions in RNA processing mechanisms may selectively affect peripheral nerves with a severity that varies according to the degree of dysfunction.^26^

## 7 IN VITRO AND IN VIVO DISEASE MODELS

The development of in vitro and in vivo models for SMARD1 has permitted great advances in the comprehension of pathogenetic mechanisms and in therapeutic studies. Two main models of SMARD1 and neuromuscular diseases in general have been developed: the stem cell model and the murine model.

### 7.1 The stem cell model

The first option for reproducing neuromuscular diseases in the laboratory originates from the isolation of human embryonic stem cells (hESCs) from the inner cell mass of the blastocyst, which was first reported in 1998.^27^ In 2006, a study by Takahashi and Yamanaka demonstrated the ability to reprogramme fibroblasts from adult mice into induced pluripotent stem cells (iPSCs), which represented an important advancement and opened the path for the use of stem cells in disease models and as a therapeutic tool. The factors Oct3/4, Sox2, c‐Myc and Klf4 act together to overwhelm skin gene expression in fibroblasts and can activate genes encoding hESC proteins, thus inducing the reprogramming of somatic cells into iPSCs.^28^ iPSCs offer great advantages compared to hESCs because these cells can be obtained from virtually any patient through a small skin biopsy sample or blood sample with no need to fertilize an embryo, thus reducing ethical issues, costs and the complexity of the procedure.^29^, ^30^

Our group provided one of the first descriptions of the generation of *IGHMBP2*‐mutated iPSCs from SMARD1 patients for in vitro studies of SMARD1.^31^, ^32^ As observed in SMA, SMARD1 iPSC‐derived motor neurons exhibit reduced survival and axonal length in culture, thus recapitulating the key features of human disease.

The limitations of the cell model are represented mainly by the inability to study the in vivo events that influence motor neuron development and degeneration, which are key aspects of SMARD1 pathogenesis.

### 7.2 The murine model

The Cox group provided the first identification of a spontaneous *ighmbp2* mutation in mice in 1998. So‐called neuromuscular degeneration *(nmd)* mice develop a disease that is similar to the human disease, with motor neuron degeneration, skeletal muscle atrophy and limb paralysis. Paralysis usually begins in the hindlimbs between the ages of 2 and 3 weeks, and then, the disease spreads to the trunk and the forelimbs. The affected mice often experience a slowdown in the progression of symptoms after that period, and most of these animals survive until the age of 14 weeks. The original phenotype of the mice was more severe, and the mice rarely survived beyond 4 weeks.^33^ The main difference between murine disease and human SMARD1 is that respiratory failure occurs at later stages in mice and is not usually the cause of death, which results apparently from dilated cardiomyopathy.^16^, ^34^

Current evidence suggests that motor neuron loss begins before the onset of clinical symptoms and that it does not affect myelin sheets in the early stages of the disease.^34^ The gastrocnemius has been found to be the most vulnerable muscle, while clinical evidence suggests that neural muscular junction (NMJ) denervation minimally affects the diaphragm, confirming the key difference in human SMARD1 patients. Furthermore, the fragmentation of the end motor plate has been detected in all analysed muscles and has been determined to be independent of their susceptibility to denervation, suggesting a role for the *ighmbp2* protein in preventing this fragmentation.^16^

## 8 THERAPEUTIC PERSPECTIVES

**Figure 1:**
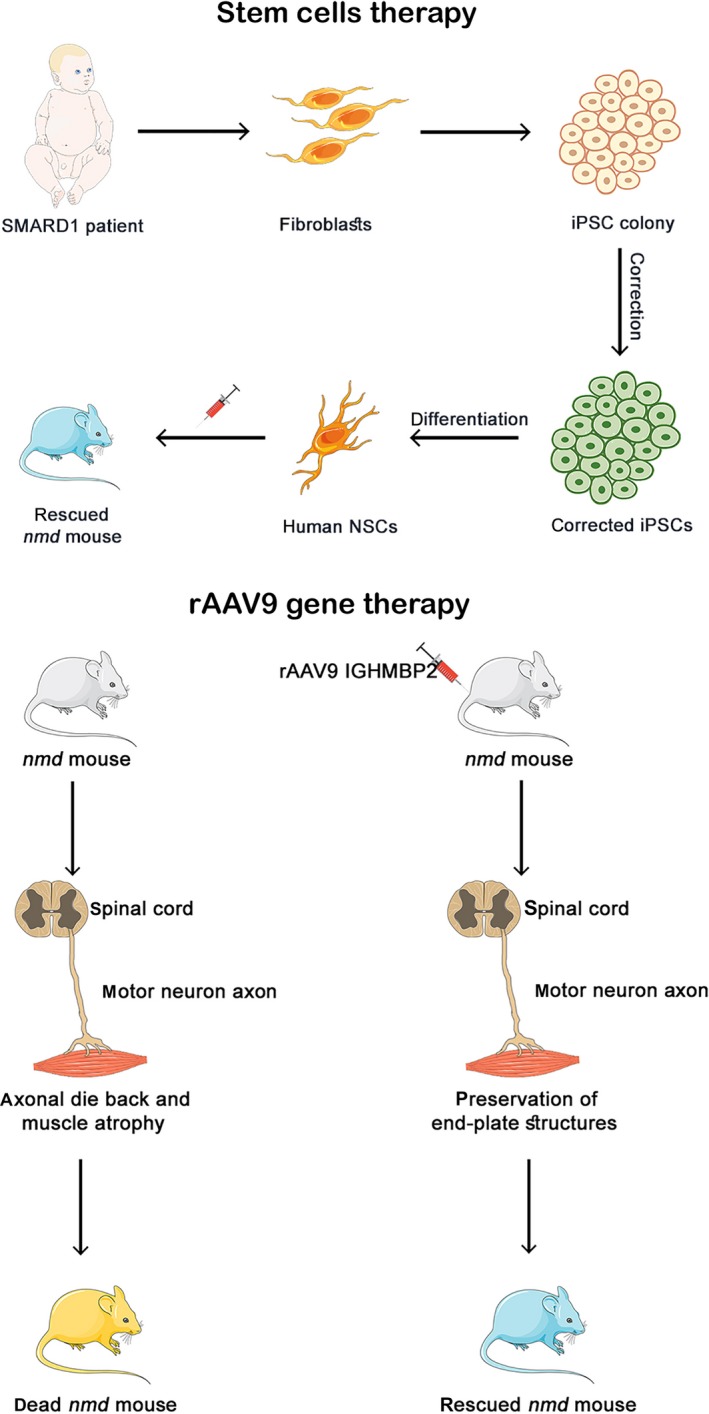
Illustrative image of preclinical therapeutic approaches for SMARD1. A, Stem cell therapy for SMARD1. Human induced pluripotent stem cells (iPSCs) can be generated from adult fibroblasts through exposure to the reprogramming factors OCT4, SOX2, NANOG, LIN28, c‐Myc and KLF4. SMARD1 spinal motor neurons (MNs) can be obtained through the differentiation of iPSCs to reproduce a disease‐specific model. The MN defects can then be corrected, and the healthy neural stem cells (NSCs) can be transplanted into an *nmd* mouse to obtain an amelioration of behavioural deficits in the animal. B, Gene therapy approach. The defective gene is replaced by the injection of recombinant adeno‐associated virus 9 (rAAV9) vectors to rescue the phenotype of the animal

Unfortunately, there is no currently available treatment for SMARD1. The collaboration between physicians and caregivers is thus fundamental to appropriately manage the critical condition of each patient, which can be a burden for all of the people living around the patient. The first important decision is whether to intubate and mechanically ventilate the child. SMARD1‐affected children have very little independence in everyday activities and often experience difficulties in feeding due to neck muscle weakness and autonomic dysfunction; thus, these children require continuous physical therapy to delay the onset of orthopaedic problems of the dorsal spine.^7^, ^10^ Discharge to home care is usually possible when the vital signs become stable and must always be the preferred option to prevent the onset of the typical complications of hospitalization, such as nosocomial infections.^10^ In recent years, many studies on the development of therapies for SMARD1 have been conducted in preclinical settings Figure 1.

### 8.1 Pharmacological studies

From a pharmacological point of view, there has been much attention paid to determining the therapeutic effect of neurotrophins in neurodegenerative diseases, particularly in those that affect motor neurons.^35^, ^36^

The Ruiz group tested the benefits of using an agonistic monoclonal antibody against trkC receptors (Mab2256) in *nmd* mice. Tyrosine kinase receptor phosphorylation plays a central role in the neuronal response to neurotrophic factors, such as brain‐derived neurotrophic factor (BDNF), ciliary neurotrophic factor (CNTF) and neurotrophin 3/4/5 (NT 3/4/5).^37^ However, the antibody failed to improve the lifespan of *nmd* mice, despite the transient amelioration of muscle strength, as demonstrated by EMG studies. Two possible explanations for the failure of this experiment are that the high haematic level of Mab2256 deregulated the trkC receptor and that the antibody did not have the required affinity for effect on the receptor.^38^

Another group headed by Krieger identified low haematic levels of IGF1 in *nmd* mice; thus, these authors investigated whether the lack of insulin‐like growth factor 1 (IGF1) is involved in motor neuron degeneration in SMARD1. The authors treated the mice with polyethylene glycol‐coupled IGF1 (PEG‐IGF1) and achieved an increase in plasma IGF1 levels, which was associated with the amelioration of neuromuscular deficits, an improvement in body weight and length, and a reduction in action potential amplitude in EMG studies compared to those of the untreated controls. Moreover, upon neuropathological examination, the nerve and muscle fibres were larger. Despite these optimistic results, no improvement in motor neuron survival was achieved, in accordance with Ruiz's results. The author's hypothesis was that the systemic PEG‐IGF1 concentration was not high enough to pass through the blood‐brain barrier, thus limiting the efficacy of this treatment.^39^

In conclusion, studies on neurotrophic factors have revealed evidence to justify future experiments to clarify whether therapeutic effects can be achieved using these substances.

### 8.2 Stem cells

Thus far, the use of stem cells as a therapeutic tool for neuromuscular diseases has been widely investigated because these cells permit the replacement of degenerating motor neurons and produce cytokines and other neuroprotective factors that can help to prevent motor neuron loss.^40^, ^41^

Our group conducted various studies aiming to develop stem cell therapies for SMARD1. In the first study, the authors observed that neural stem cells transplanted intrathecally into *nmd* mice could differentiate into three principal lineages, suggesting that the cellular environment is fundamental for stem cell differentiation. Moreover, the injected cells demonstrated the ability to differentiate into motor neurons, migrate and send their axons into the white matter and anterior horn and provided evidence of a remarkable amelioration of the neurogenic atrophy phenotype without preventing the premature death of the *nmd* mice.^42^

In a second study, we transplanted cells that had been previously differentiated into motor neurons in vitro into *nmd* mice via local delivery to determine whether the clinical phenotype could be improved. We observed that the treated *nmd* mice had higher body weights and lengths and better motility, as measured by the rotarod test. Moreover, the assessment of GFP in the engrafted neurons permitted us to determine how donor cells survive in the host; we found that the neurons exhibited good extension into the ventral roots and the corresponding white matter. Based on the results of this experiment, we concluded that motor neuron transplantation can improve clinical and neuropathological conditions in *nmd* mice.^43^

In a third study in 2014, we demonstrated the correlation between iPSC‐derived neural stem cell transplantation and improvements in clinical phenotypes and lifespan in *nmd* mice. We also observed that the number of motor neurons was significantly higher in the treated *nmd* mice than in the untreated controls. Moreover, we treated SMARD1 iPSC‐derived motor neurons and observed an amelioration of the growth and size of these cells. The hypothesis, based on the observed data, is that this improvement may be due to the production of neurotrophins (GDNF, BDNF, NT‐3 and TGF‐α) and the inhibition of HGK and GSK‐3 kinases.^31^

In conclusion, stem cells represent a valid and encouraging therapeutic perspective not only for SMARD1 but also for many other neurodegenerative diseases. The next steps that will allow the progression of this field involve other in vitro studies, followed by the in vivo testing of human stem cells to better clarify the efficacy of this therapeutic tool alone and in combination with other strategies.

### 8.3 Gene therapy

Gene therapy is the third possible approach to SMARD1 treatment, and its great advantage is that this therapy may have the potential to replace the deficient gene and restore a significant amount of protein. Recently, many gene therapy studies have been conducted in the context of neuromuscular disorders, and very encouraging results have been achieved in the treatment of SMA and in human clinical trials.^44^, ^45^ Despite these results, few studies have been performed with the same therapeutic approach in SMARD1 patients. Our group has made great efforts to test the efficacy of a gene therapy approach in an animal model of SMARD1.^32^ Our first study in 2015 showed that compared to untreated *nmd* mice, treated *nmd* mice (5 × 10^11^ viral particles administered intravenously at P1) exhibited significantly improved phenotypes in terms of lifespan (450% higher), motor function, the myofibre size of skeletal and cardiac muscles and anterior horn fibre myelination. Moreover, we generated in vitro spinal motor neurons from SMARD1 patients to verify the efficacy of this treatment in the best available model of human disease, and the obtained results were also encouraging, compared to untreated cells, the motor neurons treated with the IGHMBP2 vector exhibited increased survival, neurite length and IGHMBP2 expression.

The other important issue regarding gene therapy is its clinical translation, as three possible routes of adeno‐associated virus (AAV) vector administration have been described (intramuscular (IM), intravenous (IV) and intracerebroventricular (ICV).^46^ Although IV injection is a minimally invasive intervention and enables the viral vector to reach tissues other than those of the central nervous system, this route of administration requires more virus with an augmented rate of immunogenicity.

In two recent studies, Shababi and colleagues tested the ICV route 47 and compared this route with the IV route.^48^ In the first paper, these authors treated mice by ICV injection with 2 doses of virus (1.25 × 10^11^ or 2.5 × 10^11^ viral genomes, with the higher dose in a double volume of injection). The lower dose was associated with a significant improvement in phenotype and survival, while the higher dose was correlated with the development of hydrocephalus in both heterozygous and homozygous *nmd* mice within 1‐1.5 months of injection. The reason for this side effect is not completely defined, but it appears that both homozygous and heterozygous mice were affected, thus, it seems that the cause is related to the specific mouse genetic background rather than to the disease per se.

Moreover, in the second study, Shababi et al compared phenotypic amelioration in *nmd* mice treated with the IV and ICV administration of rAAV vectors targeting IGHMBP2 (both at the same low dose of 1.25 × 10^11^). Both treated groups showed a similar extension of lifespan (more than 200 days), weight gain and improvement in the histological features of cardiomyocytes and in cardiac fibrosis, with a significant delay in the onset of dilated cardiomyopathy compared with that of the untreated controls. However, the ICV‐treated mice gained more weight, and these mice showed significantly better rescue of hindlimb motor function, with a significant reduction in gastrocnemius contracture and a great improvement in the rotarod test performance compared to that of both the untreated controls and the IV‐treated mice. The authors concluded that the two methods of administration can be considered equally effective in the rescue of the disease phenotype of *nmd* mice due to the equivalent lifespan prolongation and cardiac improvement. ICV injection was instead correlated with a better improvement in hindlimb function.^48^ However, although the ICV and IV doses were the same, the quantity of lentiviral particles received by the CNS may have been higher after ICV delivery compared to IV delivery as ICV injections are delivered locally while IV injections are delivered systemically.

In conclusion, gene therapy seems to show encouraging results in laboratory and in vivo tests and thus requires great effort to reach the knowledge necessary to treat SMARD1.

## 9 DISCUSSION

Due to the great achievements provided by preclinical research, the field of neuromuscular diseases has quickly evolved in recent years, and some devastating diseases, such as SMA,^44^, ^45^ may finally be removed from the list of untreatable diseases. Unfortunately, SMARD1 remains an unsolved burden, probably due to its lower incidence and its complex and poorly understood pathogenetic mechanisms.

The main clinical features of this disease include neonatal onset (within the year of life), diaphragmatic paralysis and the wasting of distal limb muscles, which leads affected individuals to be completely dependent on ventilatory support and the daily supportive care of parents or caregivers.^6^, ^8^, ^9^, ^10^

The prognosis of affected patients is currently very poor,^10^, ^17^ but the advances made in the treatment of similar diseases have made it possible to study the therapeutic approaches for SMARD1. The benefits obtained by the in vitro use of neurotrophic factors and pharmacological agents are not encouraging,^38^, ^39^ but better results have been obtained with the injection of rAAV in *nmd* mice.^32^, ^48^

The pathogenetic mechanisms underlying this disease are complex and not yet completely deciphered; in fact, mutations in the same IGHMBP2 gene can determine very serious (SMARD1) or mild phenotypes (CMT). Moreover, SMARD1 itself demonstrated substantial variability in terms of age of presentation, severity of the symptoms and survival. The role of the protein in the CNS and in other systems and how the different mutations impact the pathogenesis need to be better characterized because the behaviour of the wild‐type and mutated proteins affects the strategy of application and the rate of success of the potential therapy. For example, the type of administration adopted is crucial; in fact, although ICV administration would make it possible to reach the central nervous system directly, systemic administration would have the advantage of reaching other tissues that are known to be affected by the pathogenesis. Nevertheless, the published data and the results obtained also by our group show that gene therapy represents a real potential for the treatment of SMARD1. To verify the amplitude of this potential, further studies will be required, aiming to increase the number of analysed cases, thus permitting a wider statistical analysis of the correlation between genotype, IGHMBP2 mRNA and protein levels in animal models and human iPSCs, and the clinical phenotypes observed. Future advances in the knowledge of the pathogenetic mechanisms of this disease and in gene therapy administration will also permit the development of new experimental trials to better clarify its applicability in human SMARD1 patients.

There are still many unsolved questions, such as the best route of administration, the immunogenicity of these therapies in humans, the possible side effects and the long‐term efficacy.

For the transition into the clinic, it is necessary to proceed with the validation of the method in the preclinical phase and invest in this research pathology, which, although rare, shares pathogenetic aspects with SMA and therefore also the susceptibility of treatment for SMARD1 patients.

The SMARD1 therefore represents an excellent candidate for the application of this innovative method, which has been demonstrated to be increasingly promising in the treatment of both neuromuscular and other diseases.

In conclusion, treatments for SMARD1 are not currently available, but recent preclinical therapeutic advances have laid the foundation for future solutions to this health issue, and the combination of various therapeutic possibilities that have been studied may lead to an effective therapy for SMARD1 patients.

## CONFLICT OF INTEREST

The authors declare that they have no conflict of interest.

## AUTHOR CONTRIBUTIONS

MS performed the search and wrote the manuscript; MN, AG, MT, NB and GPC contributed to manuscript writing; and SC supervised the work and wrote the manuscript. All authors read and approved the final manuscript.
